# Multi-level pooling encoder–decoder convolution neural network for MRI reconstruction

**DOI:** 10.7717/peerj-cs.934

**Published:** 2022-03-30

**Authors:** Sarattha Karnjanapreechakorn, Worapan Kusakunniran, Thanongchai Siriapisith, Pairash Saiviroonporn

**Affiliations:** 1 Faculty of Information and Communication Technology, Mahidol University, Nakhon Pathom, Thailand; 2 Department of Radiology, Faculty of Medicine Siriraj Hospital, Mahidol University, Bangkok, Thailand

**Keywords:** MRI reconstruction, Multi-level pooling, Encoder–decoder CNN, Fast MRI

## Abstract

MRI reconstruction is one of the critical processes of MRI machines, along with the acquisition. Due to a slow processing time of signal acquiring, parallel imaging and reconstruction techniques are applied for acceleration. To accelerate the acquisition process, fewer raw data are sampled simultaneously with all RF coils acquisition. Then, the reconstruction uses under-sampled data from all RF coils to restore the final MR image that resembles the fully sampled MR image. These processes have been a traditional procedure inside the MRI system since the invention of the multi-coils MRI machine. This paper proposes the deep learning technique with a lightweight network. The deep neural network is capable of generating the high-quality reconstructed MR image with a high peak signal-to-noise ratio (PSNR). This also opens a high acceleration factor for MR data acquisition. The lightweight network is called Multi-Level Pooling Encoder–Decoder Net (MLPED Net). The proposed network outperforms the traditional encoder–decoder networks on 4-fold acceleration with a significant margin on every evaluation metric. The network can be trained end-to-end, and it is a lightweight structure that can reduce training time significantly. Experimental results are based on a publicly available MRI Knee dataset from the fastMRI competition.

## Introduction

Magnetic Resonance Imaging (MRI) is a commonly used tool due to its non-invasive imaging technology. This is because the magnetic fields and radio waves are used compared with the X-ray that uses ionizing radiation. This makes the MRI a leading diagnostic tool for various disorders. However, the data acquisition process usually takes a long time inherently, which can easily exceed 30 min. This can make elderly patients feel uncomfortable when they lay still inside the MRI machine. This affects the patient throughput compared with other diagnostic tools like X-ray or CT scan. Therefore, the primary and ongoing researches in the MRI field are focused on increasing data acquisition speed.

Parallel Imaging (PI) is one of the most significant and successful developments in the MRI machine. This can accelerate the acquisition time by the number of Radio Frequency (RF) coils instead of using a single RF coil. The technique uses all information across RF coils to simultaneously record different parts of a target object rather than a standard sequential order. Then mathematics algorithms are applied for reconstruction into the final MR image (Pruessmann et al., 1999; Griswold et al., 2002). This led to a reduction in the number of phase-encoding steps during image acquisition. As a consequence, it results in a several-fold reduction in imaging time. Therefore, parallel imaging is the default option of many modern MRI scanners.

The introduction of Compressed Sensing (CS) in 2006 showed a promising breakthrough technique in MR acquisition acceleration (Candès, Romberg & Tao, 2006; Lustig, Donoho & Pauly, 2007). The CS technique accelerated the data acquisition process by acquiring less and incomplete information, an under-sampling process, required to reconstruct high-quality images. A number of acceleration folds could define the under-sampling. But the under-sampling also showed a devastating effect that was visible aliasing artifacts due to a violation of the Nyquist-Shannon sampling theorem. These artifacts must be eliminated in the reconstruction process by using an image with additional prior information. The CS could also benefit from a non-parallel MRI system.

The rapid development of machine learning approaches has been seen recently in every image processing field and also MR image reconstruction, which showed an excellent promising for the acceleration of data acquisition (Hammernik et al., 2016; Wang et al., 2016; Hammernik et al., 2018). The complexity of the deep learning technique makes it a gold standard of the machine learning. An expandable complexity of the deep learning network showed even more promising results for the MRI reconstruction at higher acceleration fold (Zbontar et al., 2018; Zhu et al., 2018). However, to achieve high-quality reconstruction results, the deep neural network requires a large-scale dataset for compensating the model complexity to prevent an over-fitting phenomenon. This can be seen as two sides of a coin of the deep learning approaches. The more complex network is, the more images are needed. Moreover, a medical dataset is harder to establish, not to mention the large-scale dataset. This is why the Facebook AI Research (FAIR) collaborated with NYU Langone Health on a research project competition that aimed to investigate the use of Artificial Intelligence (AI) to make MRI acquisition up to 10 times faster, called fastMRI. The competition provided good quality and large-scale MRI datasets with their baseline results from the deep neural network (Zbontar et al., 2018).

This paper introduces the lightweight deep convolution neural network to overcome the over-fitting problem that continually plagues the deep learning approaches in every field. The lightweight nature of the network makes it is easy to apply to other medical problems, *e.g*., medical image segmentation or another medical image type reconstruction, etc. The proposed network is based on the well-known encoder–decoder structure (Cho et al., 2014a, 2014b; Ronneberger, Fischer & Brox, 2015) that is adapted for several computer vision tasks. The conventional encoder–decoder system provides an easement to scale the complexity in both encoder and decoder modules. This makes the network very flexible to adapt and fit the variety of dataset sizes. However, it strives to reconstruct an MR image at a high accelerator. This is because the raw MR data, k-space, contains a lot of high-frequency signals, which the conventional encoder–decoder method cannot comprehend. The proposed network is added with additional modules between encoder and decoder modules to address this challenge. The extra module is called the Multi-Level Pooling module. This is also a source of the network name, Multi-Level Pooling Encoder–Decoder Net (MLPED Net). The proposed network is trained and evaluated with the fastMRI dataset (Zbontar et al., 2018).

First, a brief introduction to MR imaging techniques is provided in “Introduction” and background knowledge and related works are reviewed in “Background and Related Work”. Next, the proposed MLPED Net is explained in “Proposed Method”, followed by experiments in “Experimental Results” and the experiment’s discussion in “Discussion”. Finally, the conclusion and acknowledgment are in “Conclusion” and “Acknowledgment”, respectively.

## Background and related work

### Parallel imaging

MRI acquisitions can be a very time-consuming process since raw data in the frequency domain, k-space, are typically acquired line by line until the MR image contains a full field of view (FOV). The acquisition process uses an RF coil in an old generation of a single-coil MRI system. Then, the MR image can be obtained by applying an inverse Fourier transform function ( ${{\rm \cal F}^{ - 1}}$) to the sampled k-space data. The basis image in the Cartesian space $x \in {{\rm {\mathbb C}}^{M}}$ is connected to the sampled k-space in the Cartesian space $k \in {{\rm {\mathbb C}}^{M}}$ as below.

(1) $$k = {\rm \cal F}(x) + \alpha$$

where *α* is the measurement noise (Baert, 2007; Sriram et al., 2020).

Nowadays, modern MRI systems support multi-RF coil systems that can simultaneously scan different object parts. Each sampled k-space is modulated by its coil sensitivity to the MR signal. Each coil sensitivity is different because it depends on the distance between the object and each coil location. So the sampled MR images which already transformed will look like a set of diverse images with inhomogeneous brightness. The k-space data, which is acquired by the *i*-th coil, is described below.

(2) $${k_i} = {\rm \cal F}({S_i}x) + {\alpha _i},i = 1,2,...,N$$

where *i* is coil numbered *i*-th, *N* is a total number of coils, and *S*_*i*_ is a *i*-th coil sensitivity matrix that is measured every time before the scanning begins. Then every coiled-MR images are combined together by a reconstruction algorithm as below.

(3) $${I_{recon}} = {\rm \cal R}\left(\sum\limits_{i = 1}^N {k_i}\right)$$

where *I*_*recon*_ is a reconstructed MR image, and ${\rm \cal R}$ is a reconstruction algorithm.

### Image reconstruction

Reducing the number of k-space lines generates results as an acceleration of the acquisition time. However, when under-sampling k-space data is transformed back to an image domain results in images that contain *alias* artifacts (Hamilton, Franson & Seiberlich, 2017). Hence, modern MR scanners come with the state of the art image reconstruction algorithm. The algorithms will take under-sampled k-space or under-sample MR images, depending on each algorithm, then return a reconstructed image that contains fewer or no *alias* artifacts as a result by using redundancies in k-space data points. The reconstruction methods can be divided into two broad classes as SENSE and GRAPPA.

#### SENSE

SENSitivity Encoding for fast MRI (SENSE) is the reconstruction method that operates in the image space and utilizes encoded coil sensitivity information from every RF coil for reconstruction. Since the acceleration process creates the aliased image, which contains pixels from the fully sampled image, these pixels fold onto the same pixel in the aliased image. Therefore, the SENSE reconstructs the MR image by unfolding superimposed pixels in the aliased MR image, based on extra information from the encoded coil sensitivity (Pruessmann et al., 1999; Blaimer et al., 2004; Hamilton, Franson & Seiberlich, 2017; Baert, 2007). A matrix inversion can describe the SENSE process as below.

(4) $$\left\{ {\matrix{ {{a_1}} \cr \vdots \cr {{a_N}} \cr } } \right\} = \left\{ {\matrix{ {{s_{1,1}} \ldots {s_{1,N}}} \cr { \vdots \vdots } \cr {{s_{N,1}} \ldots {s_{N,N}}} \cr } } \right\}\left\{ {\matrix{ {{f_1}} \cr \vdots \cr {{f_N}} \cr } } \right\}$$

where *a* are pixels in the aliased MR image of coil *i*-th, *s* are the encoded coil sensitivities at coil number *i*-th and the aliased pixel number *i*-th, *f* are pixels in the fully sampled MR image, and *N* is a total number of RF coils. So a reconstructed MR image, fully sampled, can be retrieved after applying the Eq. (4) all over the aliased MR images. From the Eq. (4), it is also indicated that the SENSE process can reconstruct aliased MR images as long as an acceleration factor does not exceed a total number of RF coils. The SENSE reconstruction is as *F* = *invert*(*S*) × *A*, where *S* must be invertible or det(*S*) is not equal to zero.

#### GRAPPA

Generalized Autocalibrating Partially Parallel Acquisitions (GRAPPA) is the reconstruction method synthesizing the missing information on the under-sampling k-space. Unlike the SENSE method that is processed in the image domain. For GRAPPA, the data correction is processed first, then the reconstruction is done later (Griswold et al., 2002; Blaimer et al., 2004; Hamilton, Franson & Seiberlich, 2017; Baert, 2007). Like the SENSE method, the GRAPPA also uses the redundant k-space data. First, a central area of k-space is sampled fully to use as an Auto Calibration Signal (ACS). This is because the central information in the k-space corresponds to low spatial frequency information, which is reconstructed into the structure of the MR image. The ACS is used to calculate a GRAPPA weight (*W*) which can be expanded with a linear combination through every scan line to estimate the missing data. This can be described as a convoluted equation as below.

(5) $${K_m} = W*K$$

where *K*_*m*_ is the missing k-space data, and *K* is the known k-space data. After the missing k-space data is filled in every coils’ k-spaces, the inverse Fourier transform ( ${{\rm \cal F}^{ - 1}}$) is used for converting all k-space images back to the image space. Then, the MR images are combined into the final MR image using a sum of squares method.

#### Compressed sensing

Compressed sensing (CS) is a set of traditional signal processing methods for accelerated MR data acquisition based on a semi-random and incomplete sampling of k-space data. A final image is created through an iterative optimization process using non-Fourier transformation and thresholding of intermediately reconstructed images (Lustig et al., 2008; Geethanath et al., 2013; Donoho, 2006). A successful method of CS has three major requirements, which are:

- Incoherent Under-sampling: The artifacts caused by under-sampling k-space should be incoherent in the sparsifying transform domain.
- Sparsifying Transformation: The desired image should have a sparse representation in a known transform domain or be compressed by transform coding.
- Non-linear Iterative Reconstruction: The image should be reconstructed by a set of the non-linear method.

### Encoder–decoder network

In the last few years, machine learning methods are very popular in solving computer vision hard problems such as object classification (Redmon & Farhadi, 2017, 2018; He et al., 2017; Liu et al., 2016), medical images segmentation (Ronneberger, Fischer & Brox, 2015; Milletari, Navab & Ahmadi, 2016; Zhou et al., 2018; Gu et al., 2019), or image super-resolution (Dong et al., 2015; Li et al., 2019; Zhang et al., 2018), especially the deep learning-based approaches which are the most efficient and flexible methods. Also, many proposed techniques try to solve a reconstruction problem both in general and medical (Hammernik et al., 2018; Zhu et al., 2018; Schlemper et al., 2017; Jin et al., 2017) fields. The reconstruction problem can be seen as an inverse problem because the final result is known, but a state to transform input data into the final result is unknown. The previously mentioned advantages of the deep learning approaches make them well fits for the reconstruction problem. Because to overcome the inverse problem, the network needs to learn key features and separate them from bad signals. Then the extracted good signal features are used for formulating another network structure to restore the image. Therefore, the network needs to be flexible and easy to adapt.

From the above conditions, a type of deep neural network that stands out from others for image reconstruction is an encoder–decoder convolution neural network. The encoder–decoder is a type of deep convolution neural network that can be separated into two main parts, encoder and decoder parts (Ronneberger, Fischer & Brox, 2015; Zbontar et al., 2018). Typically the encoder–decoder network consists of a four-level structure which means four encoder and decoder modules. The encoder module acts as an image feature extractor. In each level of the encoder part, the input image features are extracted and decreased their sizes by half. Then the outputs are sent to a lower level encoder module and shipped to a decoder module at the same level for extra information. The decoder module acts as a feature selector and reconstruction unit. After receiving extracted features from a previous lower level decoder module and an exact level encoder module, the network learns to select only good signals and uses them for image reconstruction to transform the current image size into two-times larger size.

**Figure 1:**
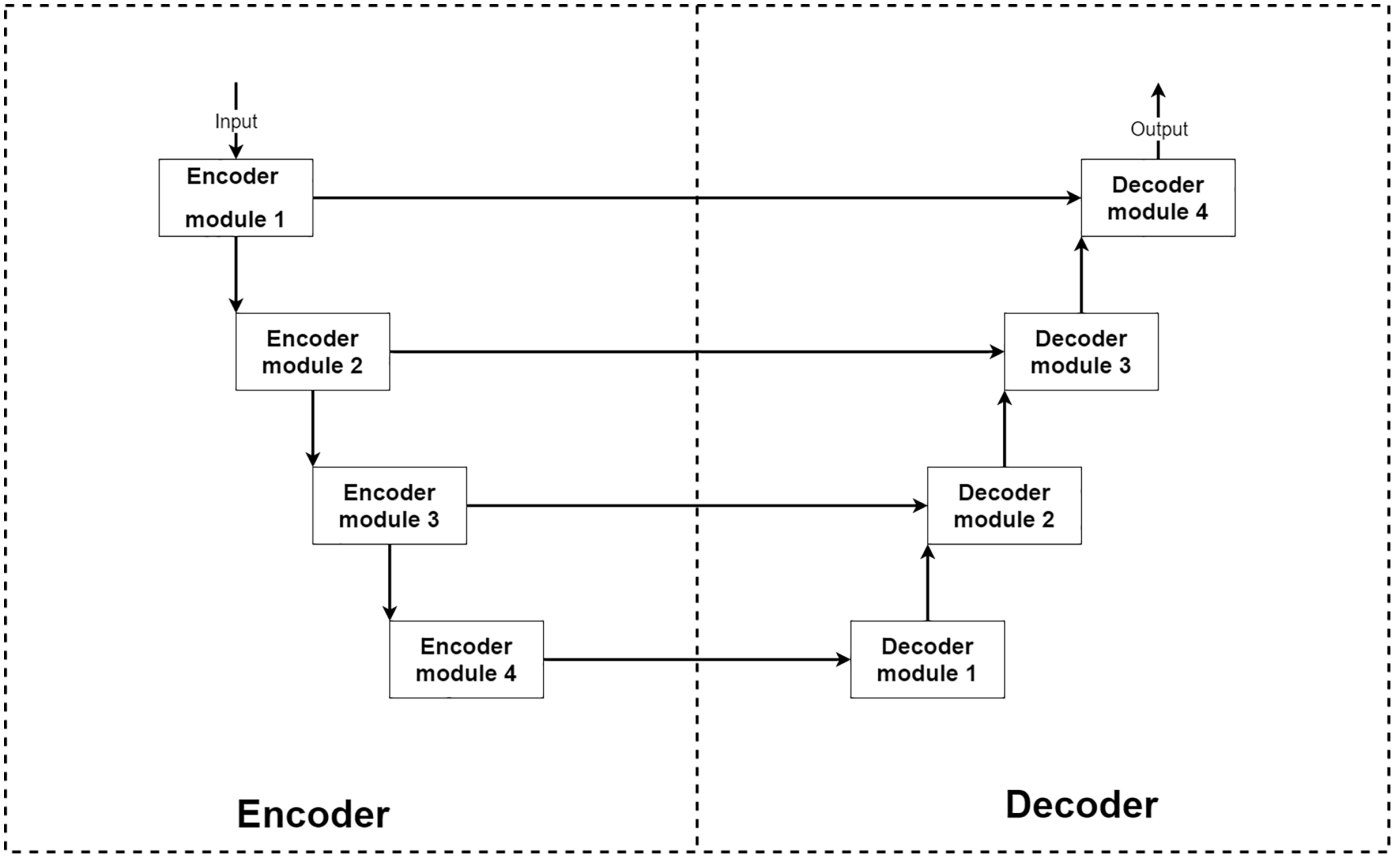
Illustration of a general structure of the encoder–decoder network.

In summary, the encoder part can decrease the input image size by eight-times smaller to extract more abstract features. The decoder part expands the input image back to the original size by using knowledge from extracted features. This makes the encoder–decoder network can learn to reconstruct the image at the abstract level. An example of the encoder–decoder network’s structure is shown in Fig. 1.

## Proposed method

### Multi-level pooling encoder–decoder net

**Figure 2:**
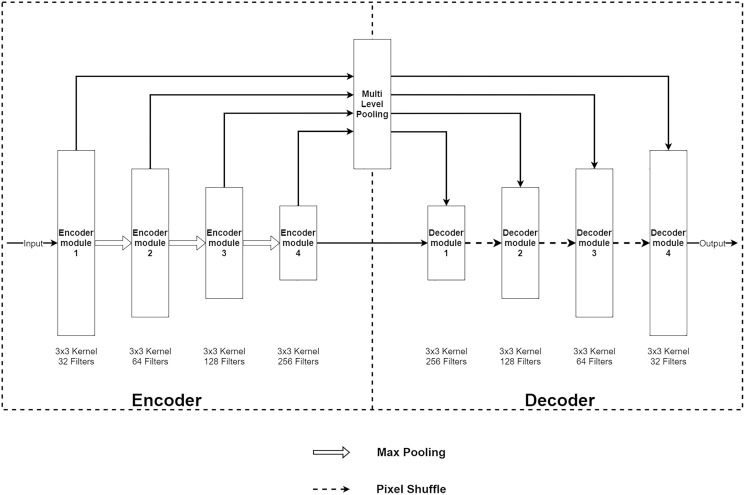
Illustration of the MLPED Net’s diagram (A) and a legend of the MLPED Net diagram (B).

The Multi-Level Pooling Encoder–Decoder Net (MLPED Net) is an encoder–decoder convolution neural network that takes an aliased MR image as an input and outputs a reconstructed MR image. Figure 2A shows an architecture of the MLPED Net that contains three major parts. First, the five-level encoder modules act as a feature extractor in consecutive order that receives all coils combined with input data. Since the under-sampled k-space from each coil depends on their coil sensitivity, it makes under-sampled MR images contain inhomogeneous brightness. To unite all multi-coil k-space data into full-resolution real-value MR image, the inverse Fourier transform ( ${{\rm \cal F}^{ - 1}}$) is applied into each coil k-space to convert back to the spatial domain. Then, a root sum-of-squares (RSS) operation combines all under-sampled MR images into a single full-resolution MR image as below.

(6) $${x_{combine}} = \left(\sum\limits_{i = 1}^N |{x_n}{|^2}\right)^{1/2}$$

where *x*_*n*_ is MR image from coil *n* and *N* is a total number of coils. The encoder module also decreases the input data size by half in every level for the abstract information extraction consecutively. This helps the network to learn the abstract feature information of an MR image. Next, the four multi-level pooling modules in four levels receive the extracted features from the encoder module and select good signals from the abstract information. Then, the selected signals are passed through the next part, *i.e*., the decoder module, at the same level. The decoder module takes the selected signals and decoded signals from the previous decoder module. Then, the abstract data type is transformed back to the normal image data. This also increases its size by half. Finally, the last decoder module outputs the reconstructed MR image at its original size.

Based on the data flow (including encoding, selecting, and decoding) of MLPED Net, it enables the network to learn to acquire as much feature data as possible in consecutive levels. Then, the selection helps the network discards irrelevance signals and keeps only good signals for the decoding to reconstruct a high-quality image. Another key benefit from the selection is it significantly decreases the network training time for a convergence and number of the network trainable parameters. This is because the selection discards noise and less impactful signals in each training epoch. Then, excellent and high-quality signals are accumulated by increasing the number of training epochs. Finally, the decoding reconstructs an MR image with fewer noises and artifacts. The whole process of the MLPED Net that contains three significant parts can be summarized as an equation below.

(7) $${x_{reconstructed}} = {\cal D}*(M*(E({x_{combined,undersampled}})))$$

where *E*, *M*, and *D* represent the encoder, the multi-level pooling, the and decoder parts, respectively. *x*_*combined*,*undersampled*_ is the single undersampled MR image and *x*_*reconstructed*_ is the reconstructed MR image.

Consequently, MLPED Net needs a small filter number and less trainable parameters to reconstruct a high-quality MR image compared with other networks. One of the critical benefits of a smaller network is that it is easy to adapt to the inference task. Usually, a machine used for inference comes with less powerful computation power when compared with the training machine. Another strength of a smaller type network is it can be trained with a smaller dataset, especially a medical image dataset that usually contains fewer training images when compared with other types of datasets and results in an over-fitting phenomenon. This also shows a promising of the filtering module type for the improving performance of the convolution neural network in reconstruction problems.

### Encoder module

**Figure 3:**
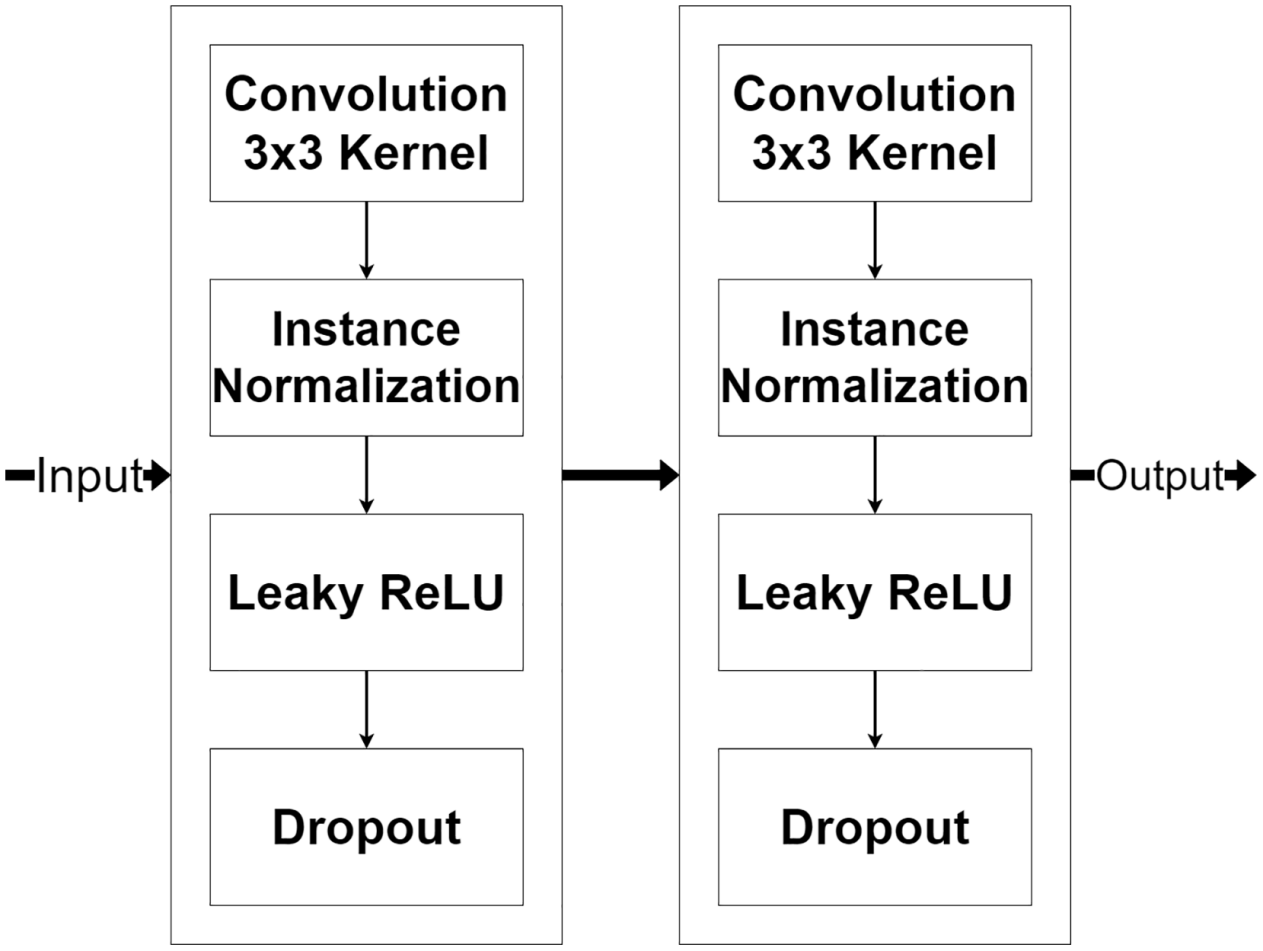
Illustration of the encoder module structure.

The encoder module is a feature extraction module of MLPED Net. MR image features are extracted in various scales, and half reduces the input image size in consecutive order. The quality of extracted features depends on the number of feature extractor filters in each level. But if the number of feature extractor filter are too many, it can make the network struggle to fit into the input data convergently. And also, the network training time is increased by the number of trainable parameters. This can be concluded as the right amount of filter number significantly affects network performance. Therefore, MLPED’s encoder module in each level is constructed based on a trade-off between the performance and the training time to prevent overfitting. Figure 3, shows the structure of each encoder module and its flow of data. It consists of two 3 × 3 convolutions, two normalizations, two Leaky ReLU activations, and two dropouts. This structure is designed for network simplification and scalability. The encoder module’s convolution with a 3 × 3 kernel contains 32 filter numbers in the first level of the network, then are increased to 64, 128, and 256 consecutively.

The pair of normalization help the module to normalize the extracted feature data because the minimum and maximum values are very different. The Leaky ReLU activation transforms the extracted feature data from the linear to the non-linear space. This lets the network learn the abstraction information of the MR image features. And the abstract information is a key to deep neural network performance. Finally, the dropouts filter out the weak extracted signals in the training process. After each encoder module, the input image’s size is decreased by half with the average pooling as shown in Fig. 2A. Figure 2A also indicates that the output from the previous encoder module is passed to the multi-level pooling module along with the lower level of the encoder module.

### Multi-level pooling module

**Figure 4:**
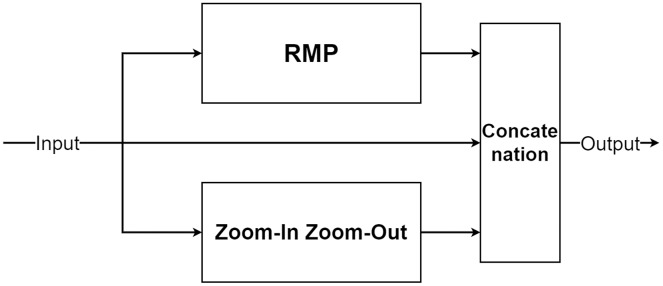
Illustration of the multi-level pooling module structure.

**Figure 5:**
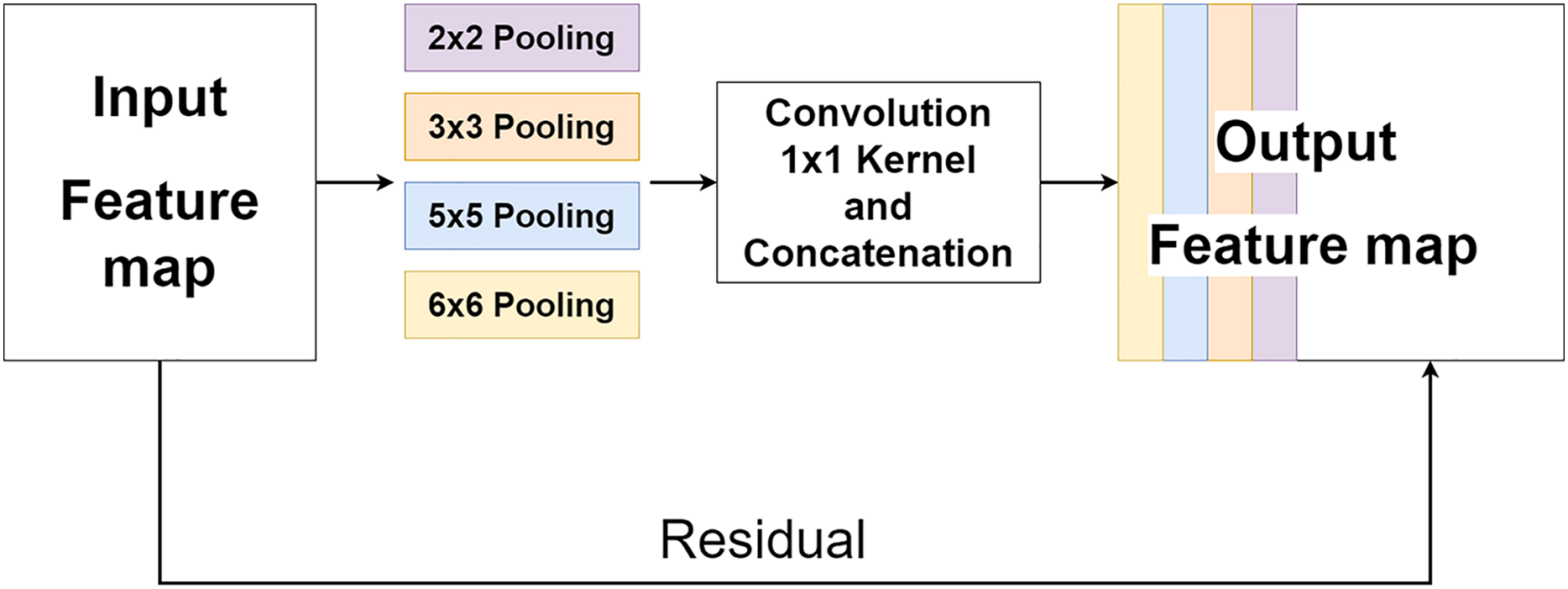
Illustration of the RMP structure.

The multi-level pooling module (MLP module) is placed between the encoder and the decoder inside MLPED Net as shown in Fig. 2A. The mlp module helps the network select a good quality of extracted features at various scales from the encoder output at the same level. And selected features are passed to the decoder module for reconstruction. In Fig. 4, it shows that the mlp module contains two major components and integrates with residual input to output a selected feature. A first component is a group of multi-size feature pooling called Residual Multi-Kernel Pooling (RMP). It acts as a gate to pick out high signal features at a specific size. The RMP was developed by Gu et al. (2019) and has been used in the medical image segmentation neural network with impactful results. Figure 5 shows the RMP structure that consists of four different size kernels: 2 × 2, 3 × 3, 5 × 5, and 6 × 6. The four kernels encode global information and output feature maps with various sizes. Then, it is followed by a 1 × 1 convolution for a dimension reduction and concatenated to the residual feature map.

**Figure 6:**
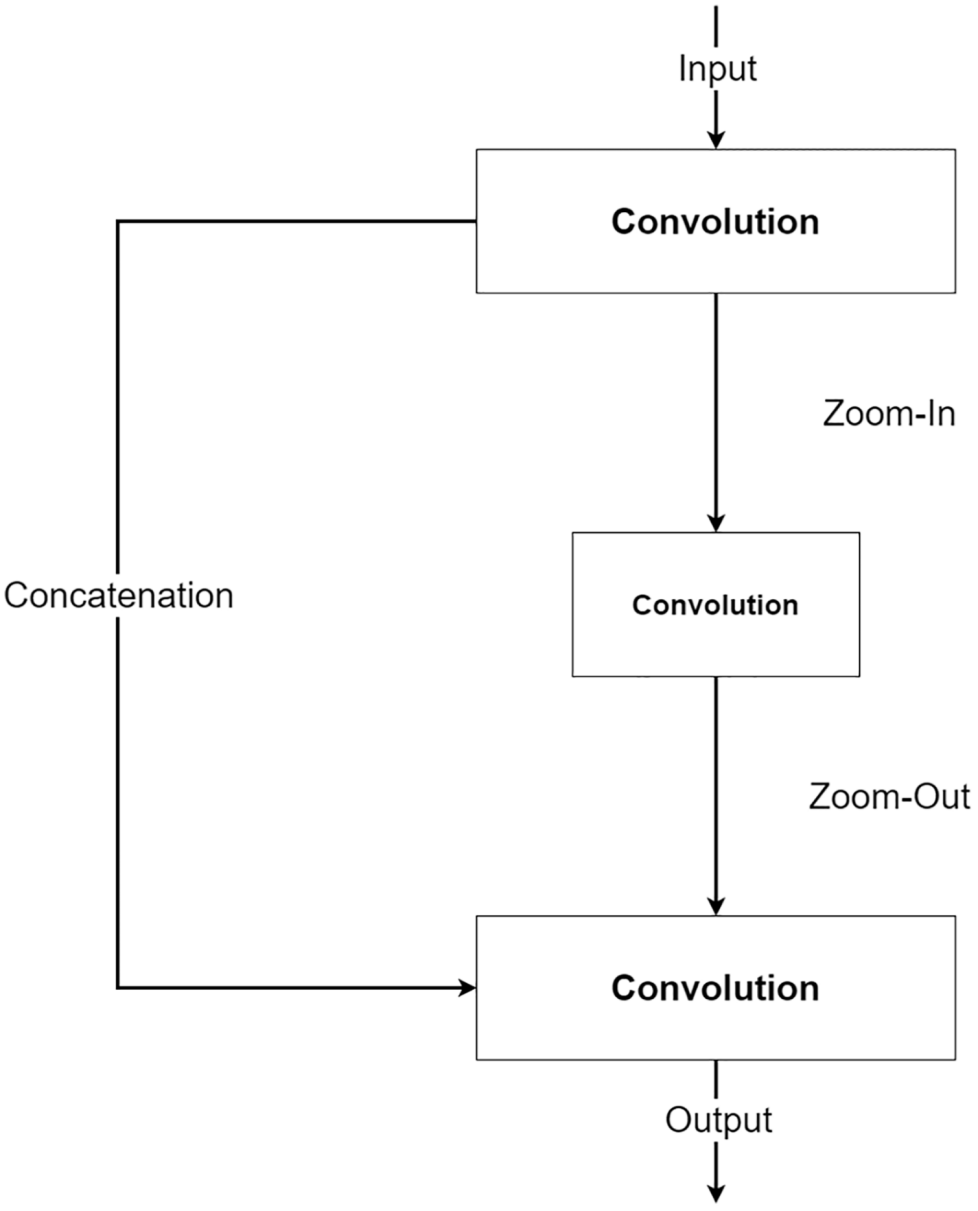
Illustration of the Zoom-In/Zoom-Out structure.

The second component is a future knowledge unit called Zoom-In/Zoom-Out. The Zoom-In/Zoom-Out is a group of three convolution layers. The Zoom-In decreases the size of features at the second convolution, where the Zoom-Out reduces the size of features mimicking the encoder module, which is then passed to the third convolution for up-sampling the feature size. The up-sampled feature mimics the decoder module explained in the following section. The Zoom-In/Zoom-Out helps the network to learn future knowledge about the decreased size and up-sampled feature, which are the essential parts of the encoder–decoder type network. A zoom factor is defined to determine how much the feature is resized. This enables the Zoom-In/Zoom-out to learn more than one level. For example, if the zoom factor equals 4, it can learn about the smaller features in two levels below it. Therefore, new ways to increase network complexity are opened by letting the network know more than two levels below or above the feature information. Figure 6 shows the diagram of the Zoom-In/Zoom-Out structure.

For the simplicity of the network, RMP is adapted by combing with the Zoom-In/Zoom-Out that uses the zoom factor of 2. So, the number of trainable parameters of the MLPED net will not exceed the number of trainable parameters of the standard encoder–decoder network by a significant margin.

### Decoder module

**Figure 7:**
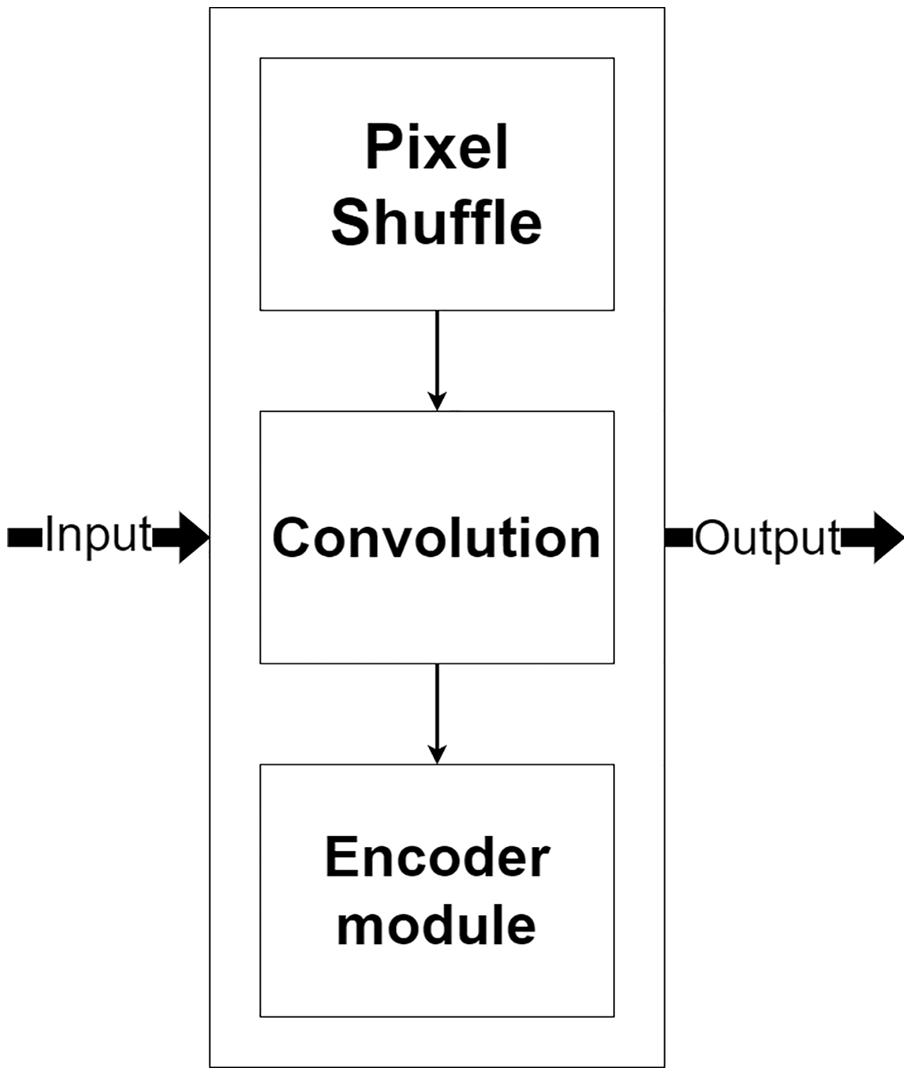
Illustration of the decoder module.

The decoder module is a part of the network that processes the selected encoded features for image reconstruction and increases their resolution. Figure 7, shows the decoder module structure that consists of two additional layers, including a pixel shuffle and a convolution, beside the encoder module. The pixel shuffle, which is a particular layer, is used for increasing the feature’s size instead of a convolution transpose or bi-linear interpolation like other techniques (Ronneberger, Fischer & Brox, 2015; Gu et al., 2019; Zhou et al., 2018). Then, the additional convolution layer and the encoder module are used for the reconstruction. One benefit of using the same encoder module structure as a part of the decoder module is that it can make the network easily reconstruct MR images from encoded features.

**Figure 8:**
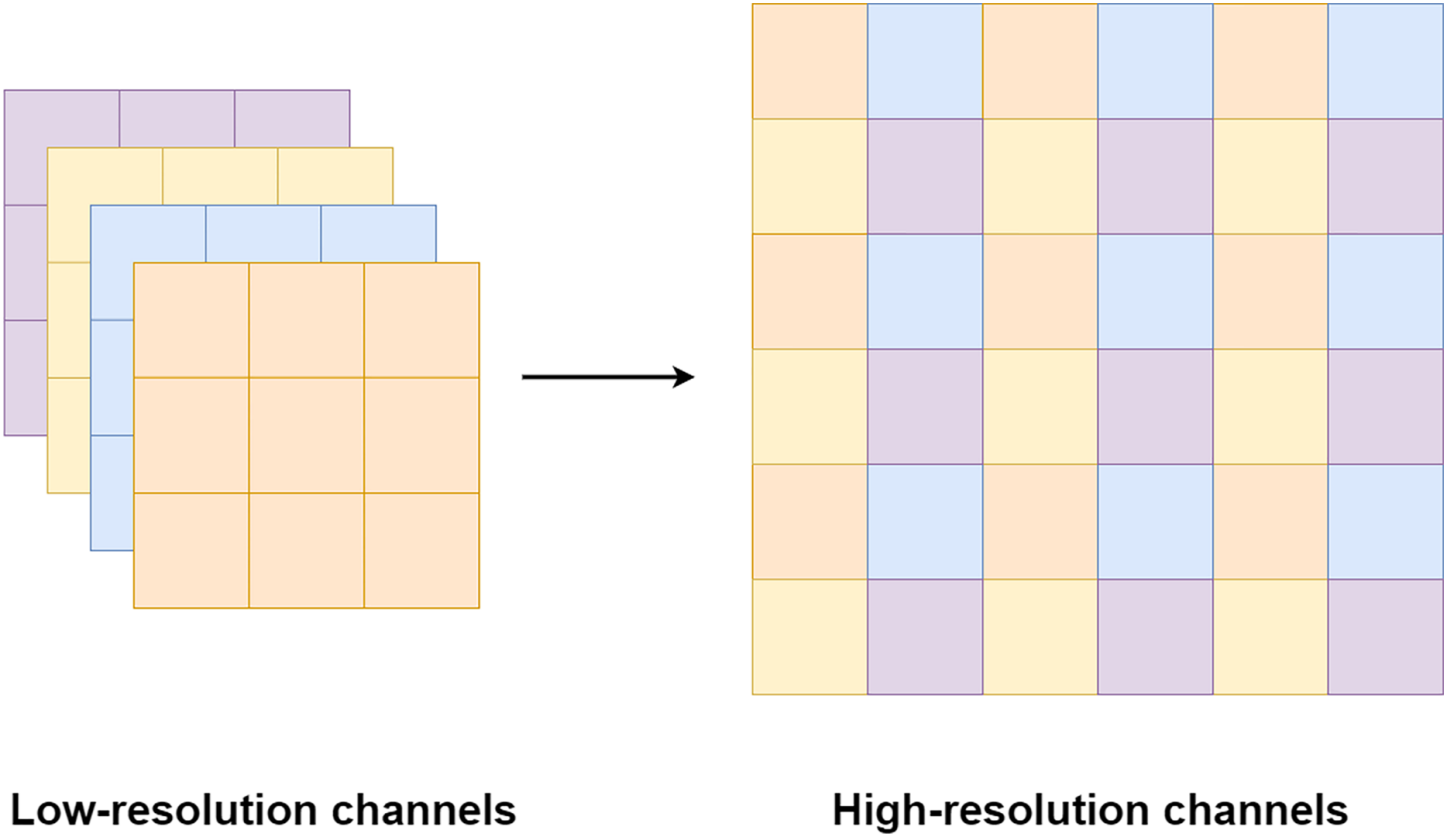
Illustration of the pixel shuffle operation.

Pixel shuffle operation is proposed by Shi et al. (2016) for an image and video super-resolution. Unlike the bi-cubic or linear interpolation that is usually used for increasing a feature size, the pixel shuffle uses additional channels from the filters to expand the feature size as shown in Fig. 8. Since the pixel shuffle operation uses information from the filters to expand the feature size, it can achieve better image reconstruction than interpolation methods or a transpose convolution. This is because the filters inside the network are trained in the training process. The network can learn to select the right signals from all channels to up-sampling the feature size. Consequently, the network that uses the pixel shuffle tends to output an image with fewer artifacts.

## Experimental results

### Details about the dataset

**Figure 9:**
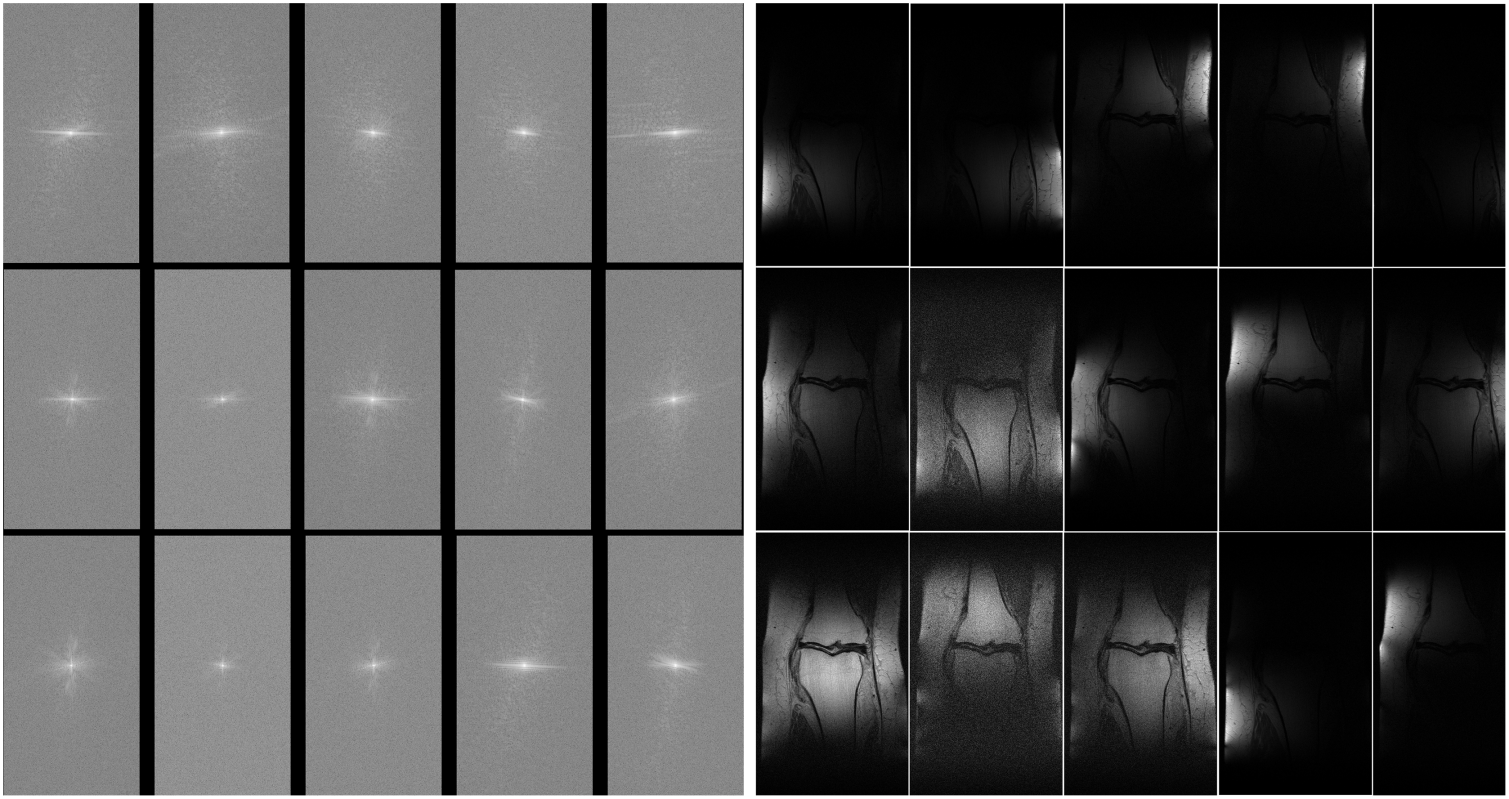
Illustration of 15 coils k-space (A) and 15 coils MR image (B).

The MLPED net was trained on a machine with 4 NVIDIA Ampere A100 40 GB GPUs using the data-parallel operation to delegate image batches for all cards. All experiments are operated on the multi-coil knee MR images from the fastMRI dataset (Zbontar et al., 2018). The fastMRI’s multi-coil knee dataset consists of raw fully sampled k-space data from 1,594 scans of four different MRI machines. Each MRI machine contains 15 coil channels that output 15 channels of k-space data. Figure 9A shows examples of 15 channels k-space of the same object and their spatial MR images after applying the inverse Fourier transform in Fig. 9B.

**Figure 10:**
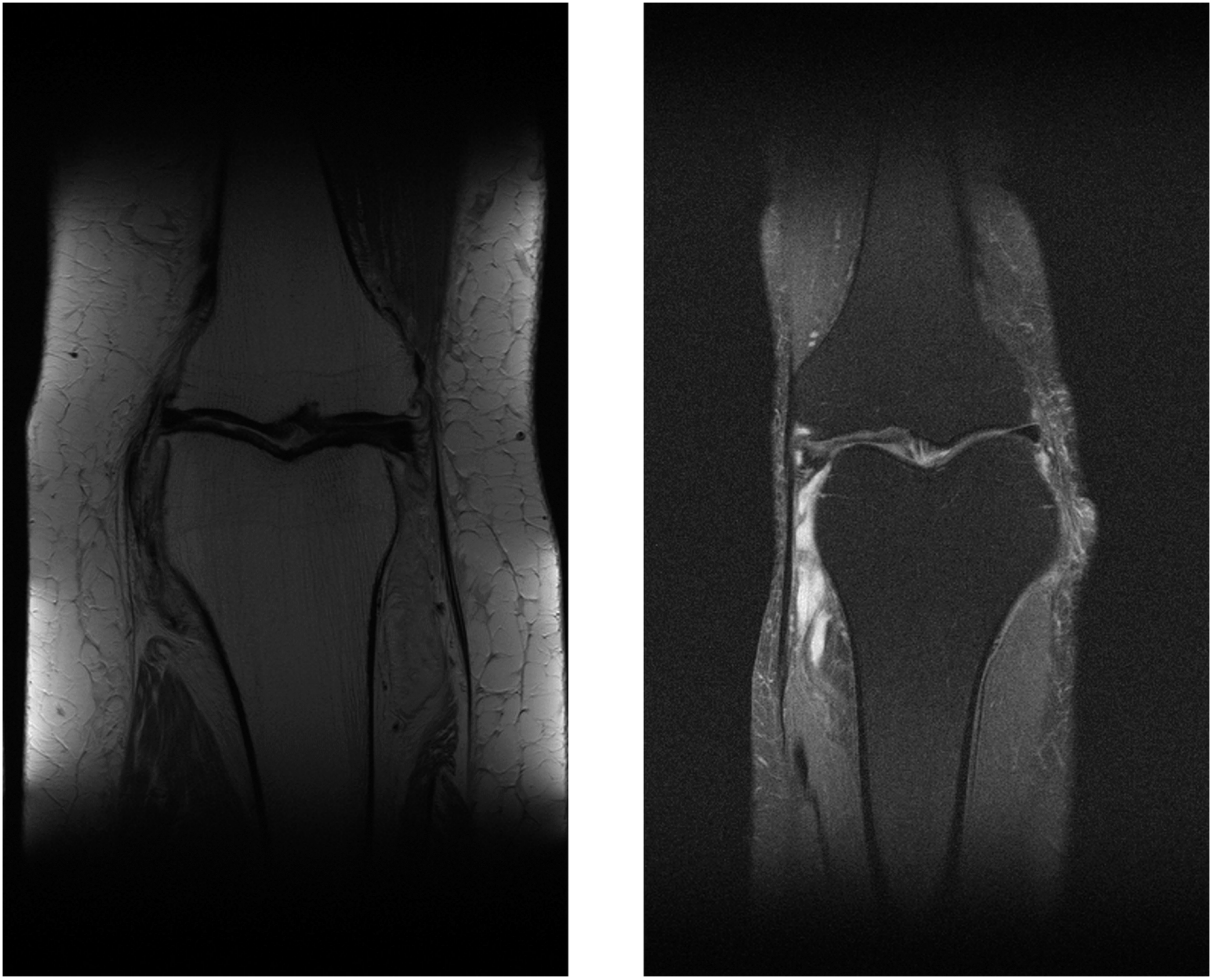
Illustration of PD MR image (A) and PDFS MR image (B).

The multi-coil knee dataset includes k-space from two pulse sequences, including coronal proton-density weighting with fat suppression (PDFS, 798 scans) and without fat suppression (PD, 796 scans). Sample MR images of PDFS and PD scan can be seen in Figs. 10A and 10B. One major challenge of using both pulse sequences is fat suppression noise in both foreground and background (Fig. 10B). This will affect the network performance. This is because the network must learn to separate noise signals from the good signals before the reconstruction process. As can be seen in the sample images, each fat suppression noise has small pixel counts compared with a knee object. However, it spreads across all over the MR image.

### Training details

**Figure 11:**
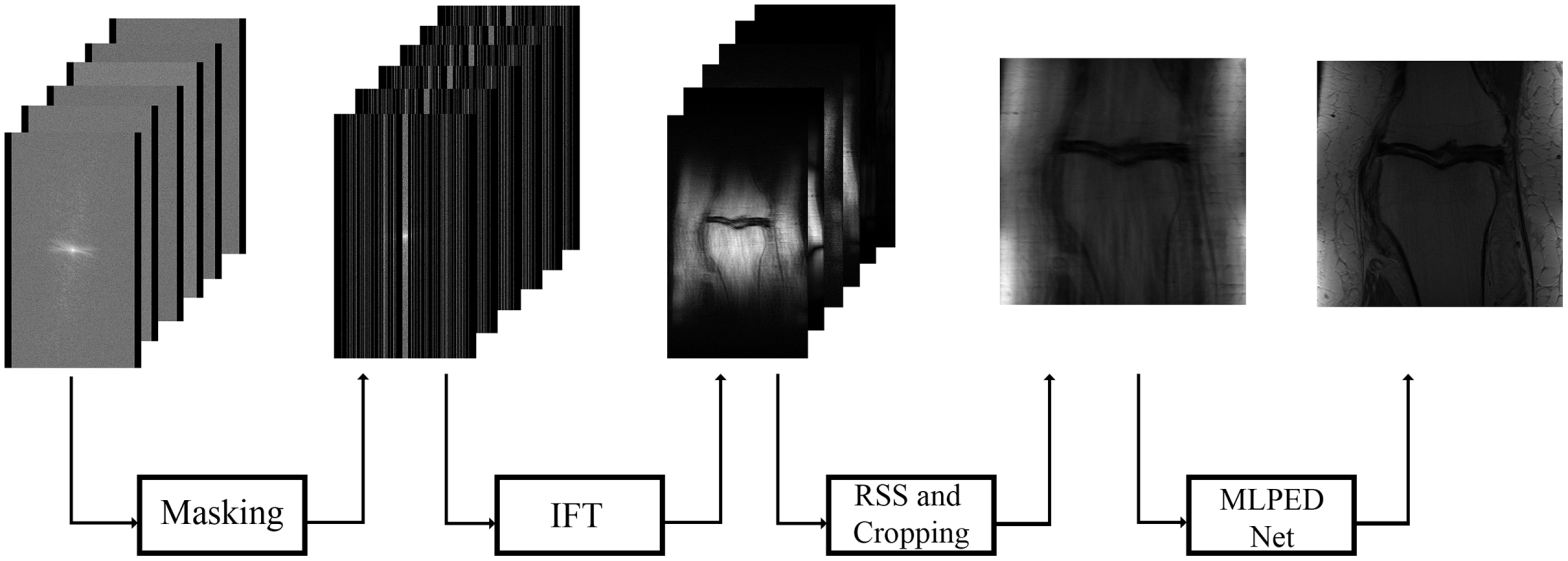
Illustration of the training process diagram.

In Fig. 11, the flow of the training process is demonstrated, starting from the preparation of input images until the step of the final reconstructed MR image. The raw multi-coil k-space data from fastMRI dataset is used as input data, but an under-sampling technique is required to simulate the MRI machine sampling acceleration. That is why a masking method is used in the fastMRI baseline process. Two types of masks are made optional to generate under-sampled k-space the way MRI machine does, including equispaced and random type with acceleration factor. The equispaced mask mimics the kind of under-sampling k-space data required by the SENSE and the GRAPPA, unlike the CS technique that requires under-sampling k-space data with random type. Masking can mimic the MRI acquisition process by dropping some of the information columns.

**Figure 12:**
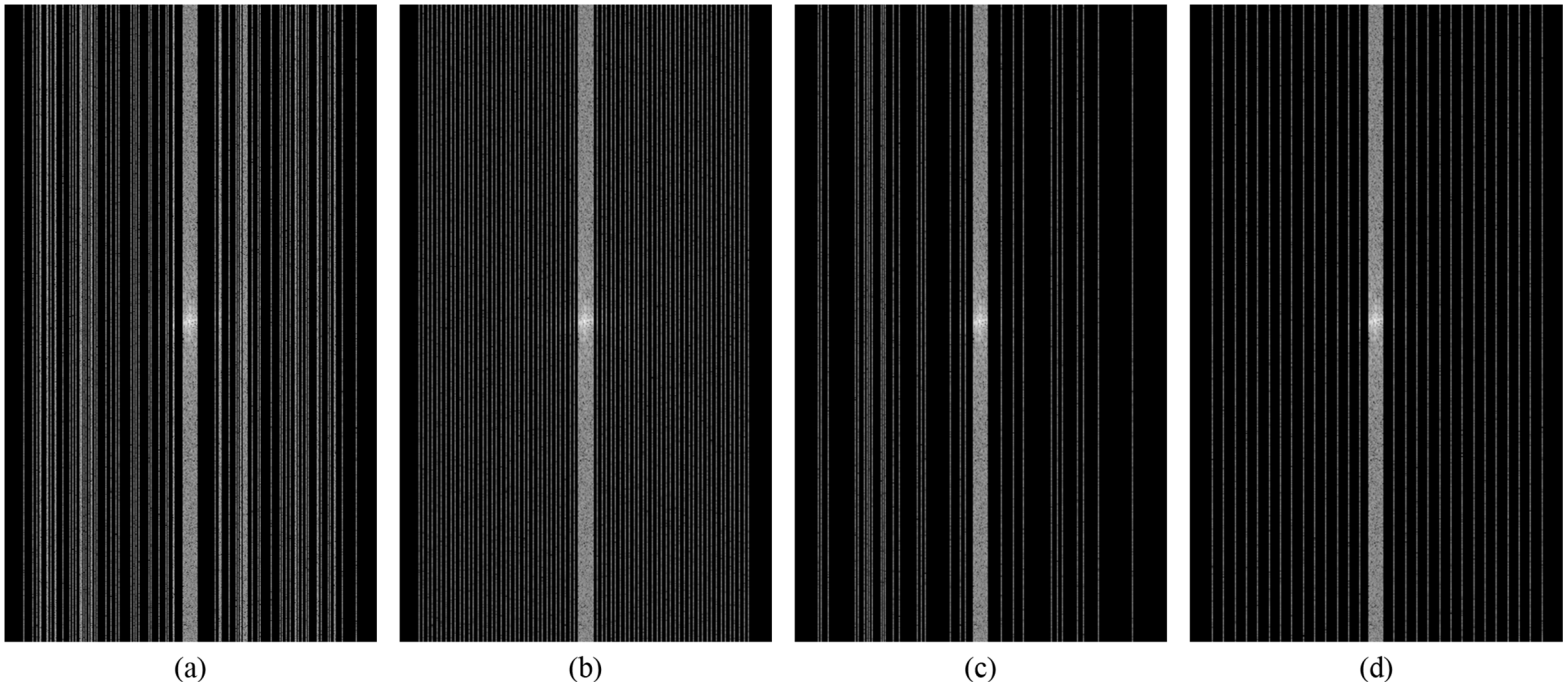
Illustration of 4-fold masked k-space with random (A) and equispaced (B) type. And illustration of 8-fold masked k-space with random (C) and equispaced (D) type.

Figures 12A–12D show examples of random and equispaced masked k-space with both 4- and 8-fold accelerations. Only random type masks with both 4- and 8-fold are used in the experiments for this research. So, the MLPED net aims to use the type of under-sampling k-space data the CS technique used. Then the masked k-space data are transformed back to the spatial domain by the inverse Fourier transform, resulting in aliased multi-coil knee MR images. Before the aliased knee MR images are passed into the MLPED Net, the RSS technique combines multi-coil aliased MR images into a single aliased MR image with a homogeneous brightness level. Then RSS images are cropped with only the center part of a size 320 × 320 pixels. After the training process is finished, the MLPED Net will output a reconstructed MR image to mimic the fully-sampled MR image from the MRI machine.

The MLPED Net was trained with the RMSProp optimizer to minimize *L*1 losses below.

(8) $$M({x_{recon}},{x_{gt}}) = ||{x_{recon}} - {x_{gt}}{||_1}$$

where *x*_*recon*_ is the reconstructed MR image and *x*_*gt*_ is the ground truth MR image. The network was trained for 100 epochs with a learning rate of 0.001. The data-parallel is also utilized to distribute the input data across all GPUs.

### Results

Experimental results are shown in Table 1, which are comparisons between the proposed network with the fastMRI baseline results using three metrics: (1) normalized mean squared error (NMSE), (2) peak signal-to-noise ratio (PSNR), and (3) structural similarity (SSIM). The table indicates that the proposed network outperforms the fastMRI baseline network in every category, including pulse sequences and 4- and 8-fold accelerations. Table 2 compares all metrics across five training attempts in both 4- and 8-fold accelerations to test the proposed network’s stability. The results are consistent on all training attempts with minimal deviations.

**Table 1:** Comparison between the proposed method and the fastMRI baseline network performance for the multi-coil knee task on the validation dataset.

Model	Acceleration	Channels	NMSE		PSNR		SSIM	
			PD	PDFS	PD	PDFS	PD	PDFS
MLPED Net	4-fold	32	0.00496	0.01094	37.99	36.52	0.9316	0.8671
fastMRI network		256	0.00540	0.01120	37.58	36.39	0.9287	0.8655
MLPED Net	8-fold	32	0.00938	0.01603	35.19	34.77	0.8939	0.8294
fastMRI network		256	0.01200	0.01810	34.12	34.23	0.8915	0.8286

**Table 2:** Stability evaluation of the proposed network based on five training attempts with the validation set of the fastMRI dataset.

Acceleration	Channels	NMSE		PSNR		SSIM	
		PD	PDFS	PD	PDFS	PD	PDFS
4-fold	32	0.00496	0.01094	37.99	36.52	0.9316	0.8671
		0.00550	0.01138	37.52	36.32	0.9271	0.8643
		0.00558	0.01147	37.46	36.29	0.9268	0.8640
		0.00536	0.01128	37.64	36.37	0.9283	0.8652
		0.00548	0.01141	37.54	36.32	0.9272	0.8643
Average		0.00522	0.01118	37.77	36.42	0.9294	0.8657
8-fold	32	0.00938	0.01603	35.19	34.77	0.8939	0.8294
		0.00958	0.01634	35.09	34.72	0.8925	0.8286
		0.00964	0.01634	35.07	34.71	0.8921	0.8285
		0.00957	0.01618	35.10	34.72	0.8925	0.8287
		0.00959	0.01621	35.09	34.72	0.8926	0.8286
Average		0.00958	0.01628	35.09	34.72	0.8926	0.8286

**Figure 13:**
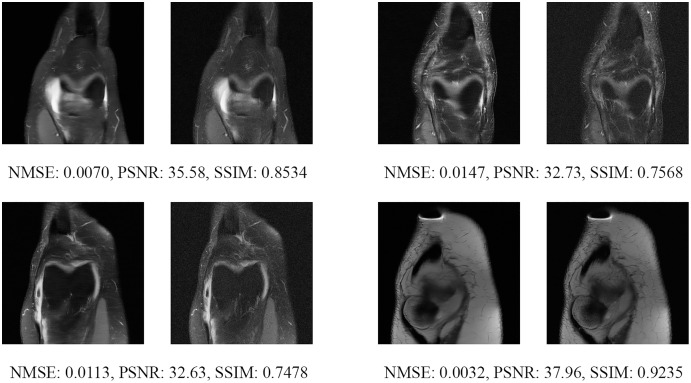
Four examples of 4-fold reconstructed images with the MLPED Net at slice number 10 of 32 slices along with evaluated metrics. The left image represents the reconstructed image, and the right means the ground truth.

**Figure 14:**
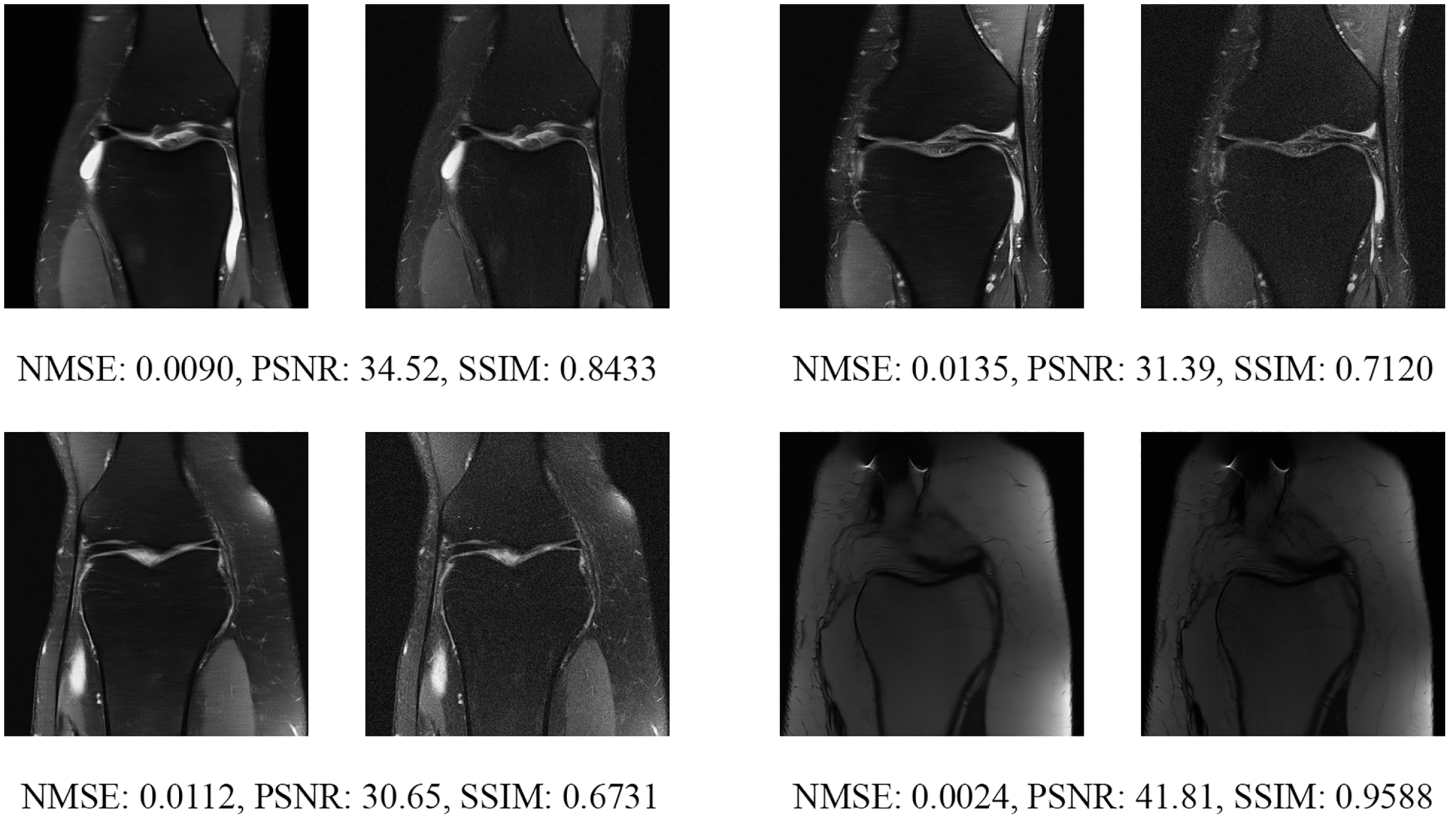
Four examples of 4-fold reconstructed images with the MLPED Net at slice number 15 of 32 slices along with evaluated metrics. The left image represents the reconstructed image, and the right means the ground truth.

**Figure 15:**
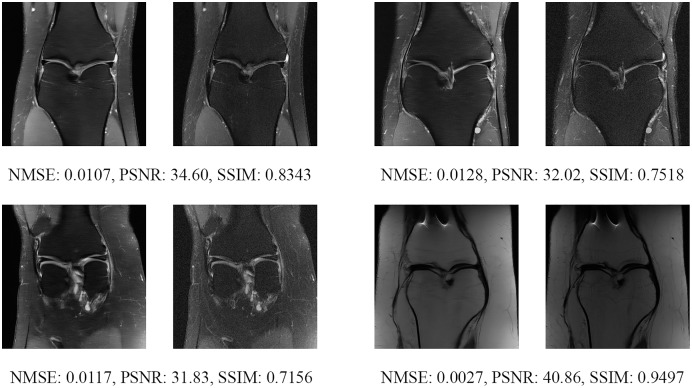
Four examples of 4-fold reconstructed images with the MLPED Net at slice number 20 of 32 slices along with evaluated metrics. The left image represents the reconstructed image, and the right means the ground truth.

**Figure 16:**
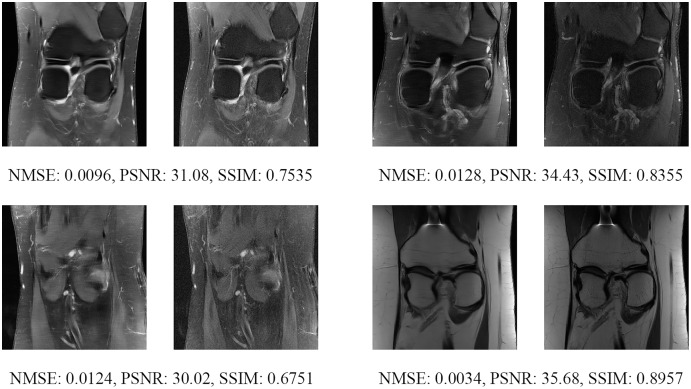
Four examples of 4-fold reconstructed images with the MLPED Net at slice number 25 of 32 slices along with evaluated metrics. The left image represents the reconstructed image, and the right means the ground truth.

**Figure 17:**
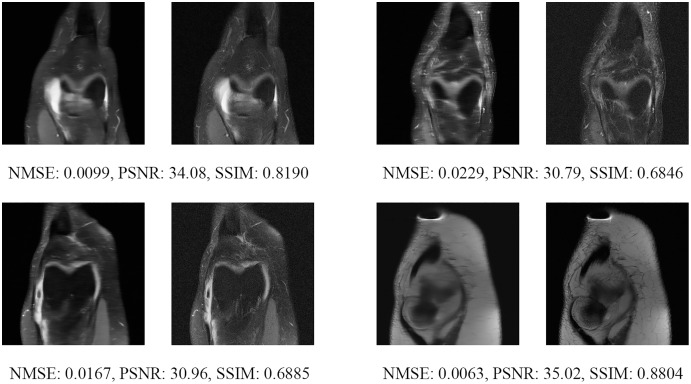
Four examples of 8-fold reconstructed images with the MLPED Net at slice number 10 of 32 slices along with evaluated metrics. The left image represents the reconstructed image, and the right means the ground truth.

**Figure 18:**
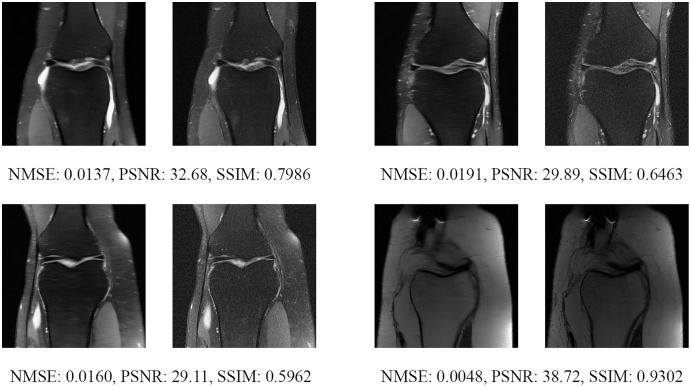
Four examples of 8-fold reconstructed images with the MLPED Net at slice number 15 of 32 slices along with evaluated metrics. The left image represents the reconstructed image, and the right means the ground truth.

**Figure 19:**
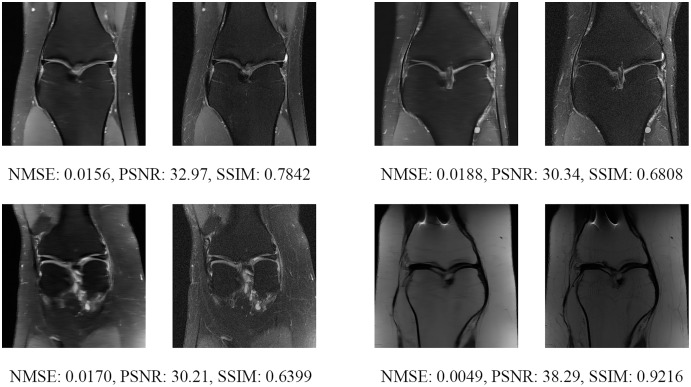
Four examples of 8-fold reconstructed images with the MLPED Net at slice number 20 of 32 slices along with evaluated metrics. The left image represents the reconstructed image, and the right means the ground truth.

**Figure 20:**
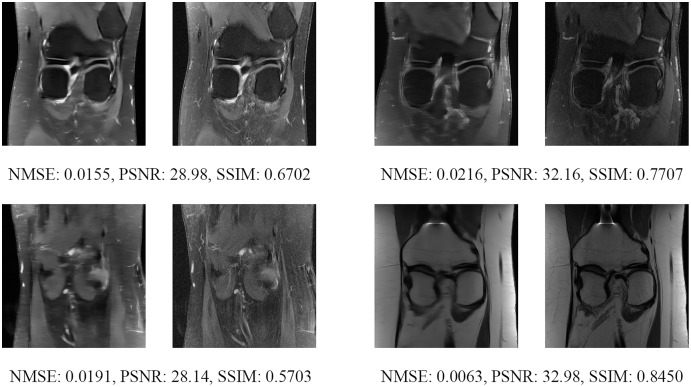
Four examples of 8-fold reconstructed images with the MLPED Net at slice number 25 of 32 slices along with evaluated metrics. The left image represents the reconstructed image, and the right means the ground truth.

Since the fastMRI competition only provides ground-truth images for the training and validation datasets, the following reconstructed images are based only on the fastMRI’s validation dataset. Examples of reconstructed MR images are shown in Figs. 13–20 for 4- and 8-fold accelerations with both pulse sequences at four difference slices respectively. Figures 13–20 also show the ground truth MR images, fully-sample MR images, with the evaluated metrics for a comparison.

**Figure 21:**
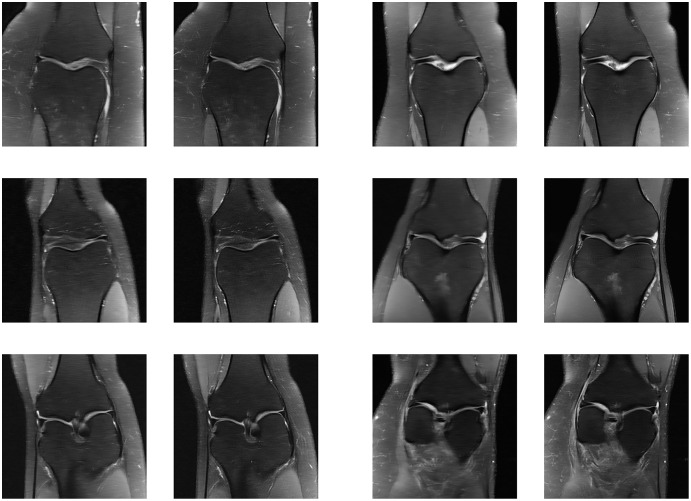
Six examples of the reconstructed images on the knee multi-coil test dataset. Each example shows a comparison between the MLPED Net on the Left and the fastMRI pre-trained U-Net competition version on the Right.

Another way to measure the proposed network performance is to test the unknown dataset. In that case, the fastMRI’s multi-coil knee test dataset is used for measuring the proposed network performance by comparing it with the public fastMRI pre-trained network. The public fastMRI pre-trained network is based on the traditional U-Net structure with the 256 filter numbers in the first layer, as shown in Table 1. Figure 21 show comparisons between the proposed network and the publicly available fastMRI pre-trained network. Unfortunately, the fastMRI’s multi-coil knee test datasets do not have ground-truth images. So the evaluation metrics cannot be measured.

## Discussion

The experimental results show promise on both scenarios of 4- and 8-fold accelerations. In Table 1, it is evident that the MLPED Net outperforms the fastMRI baseline network in every evaluation metric, especially on the NMSE. The MLPED Net exceeds the fastMRI baseline network on both 4- and 8-fold accelerations with fewer channels by eight times.

With a significantly smaller network’s size, the MLPED Net is easy and takes a smaller amount of time to train the network when compared with the fastMRI baseline network, as shown in Table 3. The table also indicates that the MLPED Net contains fewer trainable parameters when compared with the fastMRI baseline network (27 times fewer), even though they are both based on the same encoder–decoder neural network type. Both networks are trained with the same 973 training images per epoch for 100 epochs. This finally leads to a significantly less training time per epoch. One significant benefit of a smaller neural network is preventing an over-fitting phenomenon on a smaller dataset. This helps a lot with the problems that do not have large datasets. Therefore, the MLPED Net can adapt to other issues quickly.

**Table 3:** Comparison between the proposed method and the fastMRI baseline network on the training process’s performance with the 973 training images per epoch for 100 epochs.

Model	Channels	Number of trainable parameters (million)	Training time per epoch (minute)
MLPED Net	32	8	14
fastMRI network	256	214.16	120*

**Note:**

* Measured by running the fastMRI public baseline source code.

One key advantage of the deep neural network, compared with the traditional machine learning methods, is achieving a higher performance on the unknown dataset. This is because, typically, the deep neural networks contain a lot of trainable parameters with complex layer graphs that make them more flexible to adjust to the unknown data. Since the MLPED Net includes a small number of trainable parameters and a streamlined structure, a test on the anonymous dataset is necessary to measure the network performance to confirm that the MLPED Net exceeds or stays at the same level as the fastMRI baseline network.

However, the fastMRI does not provide ground-truth images for the test dataset, so the performance metrics cannot be measured on the testing dataset. And only a competition version of the pre-trained network is provided by the fastMRI, which uses both the train and validation datasets to form a large-scale dataset for a training process. That is also a different version from the published version from the Zbontar et al. (2018). This is beyond the scope of this research that has to use the validation dataset for measuring inside the training process. Therefore, examples of reconstructed images that are demonstrated in Figs. 21A–21F are comparisons between the MLPED Net performance on the unknown dataset with the normal training process and the competition version of the fastMRI pre-trained network with the combined dataset training process.

## Conclusion

In this paper, the MLPED Net is introduced for an MRI reconstruction. The MRI reconstruction is a challenging problem due to the complexity of low and high-frequency information acquired during the acquisition process. Then the frequency information is transformed back to spatial domains to form up the MR images. But the acquired frequencies are not entirely received due to the acceleration process inside the MRI machine. Therefore, the transforming process required extra techniques to make sure that the final MR image is close to the original object as much as possible. The proposed MLPED Net could address this transforming process by using the strength of the deep neural network. A structure of the MLPED Net is based on the well-known encoder–decoder type of network that performs well on the reconstruction or enhancement problems. Besides the traditional encoder–decoder system, the MLPED Net also includes multi-layer pooling between encoder and decoder modules in every layer. This helps the network eliminate the irrelevance information and could result in a faster training process with a significantly smaller network size when compared with the existing encoder–decoder network with a larger size. Since an under-sampled MR image contains a lot of noise and artifacts, the network that can discard noise efficiently can reconstruct a better result image.

Reconstruction results of the proposed network on a multi-coil knee validation dataset outperform the U-net baseline network in every metric, including NMSE, PSNR, and SSIM. From the reconstructed images, it can be seen that even tiny details look similar to ground truth images. These show the strength of the proposed MLPED Net, which can preserve small amounts of information that are usually hard to reconstruct.

## Supplemental Information

10.7717/peerj-cs.934/supp-1 Supplemental Information 1 Source code for training a new model with developed architecture, under the same framework of FastMRI schema.

10.7717/peerj-cs.934/supp-2 Supplemental Information 2 MLPED related codes covering the whole project including the mlped.

## Additional Information and Declarations

### Data Availability

The following information was supplied regarding data availability: The code is available in the Supplemental Files. The dataset used in this research project is available from the original website at https://fastmri.med.nyu.edu/.
